# Nucleotide Sequence Variation within the PI3K p85 Alpha Gene Associates with Alcohol Risk Drinking Behaviour in Adolescents

**DOI:** 10.1371/journal.pone.0001769

**Published:** 2008-03-12

**Authors:** Sylvane Desrivières, Kristina Krause, Anne Dyer, Josef Frank, Dorothea Blomeyer, Mark Lathrop, Karl Mann, Tobias Banaschewski, Manfred Laucht, Gunter Schumann

**Affiliations:** 1 Section of Addiction Biology, Interdisciplinary Research Group “Addiction” and NIHR-Biomedical Research Centre, Institute of Psychiatry, King's College, London, United Kingdom; 2 Molecular Genetics Laboratory, Department of Addiction Medicine, Central Institute of Mental Health, Mannheim, Germany; 3 Centre National de Genotypage, Evry, Paris; 4 Department of Child and Adolescent Psychiatry and Psychotherapy, Central Institute of Mental Health, Mannheim, Germany; 5 Department of Psychosomatic Medicine, Central Institute of Mental Health, Mannheim, Germany; 6 Division of Clinical Psychology, Department of Psychology, University of Potsdam, Germany; Charité University Medicine, Germany

## Abstract

**Background:**

While the phosphatidylinositol 3-kinase (PI3K)-dependent signaling pathway is typically known to regulate cell growth and survival, emerging evidence suggest a role for this pathway in regulating the behavioural responses to addictive drugs.

**Methodology/Principal Findings:**

To investigate whether PI3K contributes to patterns of risky alcohol drinking in human, we investigated genetic variations in *PIK3R1*, encoding the 85 kD regulatory subunit of PIK, in 145 family trios consisting of 15–16 year old adolescents and their parents. Screening for mutations in exons, exon-intron boundaries and regulatory sequences, we identified 14 single nucleotide polymorphisms (SNPs) in the *PIK3R1* gene region from exon 1 to the beginning of the 3′ untranslated region (UTR). These SNPs defined haplotypes for the respective *PIK3R1* region. Four haplotype tagging (ht)SNPs (rs706713, rs2302975, rs171649 and rs1043526), discriminating all haplotypes with a frequency ≥4.5% were identified. These htSNPs were used to genotype adolescents from the “Mannheim Study of Risk Children” (MARC). Transmission disequilibrium tests in these adolescents and their parents demonstrated sex-specific association of two SNPs, rs2302975 and rs1043526, with patterns of risky alcohol consumption in male adolescents, including lifetime prevalence of drunkenness (p = 0.0019 and 0.0379, respectively) and elevated maximum amount of drinking (p = 0.0020 and 0.0494, respectively), as a measure for binge drinking pattern.

**Conclusions/Significance:**

Our findings highlight a previously unknown relevance of *PIK3R1* genotypes for alcohol use disorders and might help discriminate individuals at risk for alcoholism.

## Introduction

About 76.3 million people worldwide have diagnosable alcohol use disorders, according to the World Health Organization (WHO; Global Status Report on Alcohol 2004), placing alcohol-response related behaviours among the most common psychiatric disorders. Harmful drinking frequently originates during adolescence, which increases the risk of developing alcohol-related problems later during adulthood [1] and might facilitate the development of alcohol dependence.

Twin and adoption studies have demonstrated that genetic factors significantly contribute to the development of addiction, including alcohol addiction [2], [3]. Despite strong evidence of genetic effects for vulnerability to alcohol dependence, linkage-based analyses failed to identify major gene effects. Association studies have led to the identification of several gene variants that might potentially predispose to alcohol dependence, however the only genes that have been conclusively found to influence alcohol consumption and dependence are genes involved in alcohol metabolism [reviewed in 4], [5]. Difficulty to identify genes that contribute to the risk of alcohol dependence likely reflects the fact that multiple genes with modest effects contribute to the development of this disorder. In addition, age- and gender-specific genetic differences might be critical in the aetiology of alcoholism [4].

The glutamatergic neurotransmission system is a major site of action for drugs of abuse, including alcohol [6]. Involvement of ionotropic glutamate receptors in the pathophysiology of alcoholism has been established [7] and growing evidence indicates that metabotropic glutamate (mGlu) receptors also play a particularly important role in mediating the behavioural effects of alcohol. Administration of an mGluR5 antagonist in rodent reduces self-administration of ethanol [8]–[12] and abstinence-induced increases in ethanol intake [9].

The signalling pathways underlying the pathophysiological changes associated with alcoholism are only starting to be understood. Recent data indicate that coupling of both type of glutamate receptors to the phosphatidylinositol 3-kinase (PI3K) pathway regulates synaptic plasticity [13]–[17]. Interestingly, studies using animal models for addiction revealed hints for involvement of PI3K in behavioural responses to addictive drugs. PI3K was found to be necessary for behavioural sensitisation to cocaine in rats [18], and for normal sensitivity to the acute intoxicating effects of ethanol in flies [19].

Taking these findings into consideration, we hypothesized that PI3K might contribute to human alcohol abuse. To test this, we performed a genetic screen to identify potentially functional polymorphisms within the *PIK3R1* gene associating with specific patterns of alcohol consumption in adolescents.

## Methods

### Participants

Participants were recruited from the “Mannheim Study of Risk Children” (MARC) [20], a longitudinal study that follows children at risk for later psychopathology from birth to adolescence. The initial sample comprised 384 children of predominantly (>99.0%) European descent born between 1986–88. Only firstborn children with singleton births and German-speaking parents were enrolled in the study. Furthermore, children with severe physical handicaps, obvious genetic defects, or metabolic diseases were excluded. The current investigation included 145 trios (77 male, 68 female adolescents and their parents) for whom complete genotype and phenotype data were available. Demographic and clinical characteristics of the adolescents are given in Table 1. The adolescents were in average 14.99±0.37 year old. Loss of subjects was not selective with regard to sex or risk group, and the excluded adolescents did not differ from the included in terms of drinking behavior. Written informed consent or (in the case of adolescents) assent was obtained from all individuals when they were in a state of full legal capacity. The study was approved by the ethics committees of the University of Heidelberg.

**Table 1:** Demographic and clinical characteristics in male and female adolescents

	Males (n = 77)	Females (n = 68)	Total (n = 145)
Age: mean (SD)	15.0 (0.3)	14.9 (0.4)	15.0 (0.4)
IQ: mean (SD)	104.6 (14.2)	105.5 (14.9)	105.0 (14.5)
Psychosocial risk score^1^: mean (SD)	1.34 (1.64)	1.44 (1.65)	1.39 (1.64)
Obstetric risk score^2^: mean (SD)	1.16 (1.08)	0.97 (0.98)	1.07 (1.03)
Lifetime drinking: n (%)	58 (75.3)	51 (75.0)	109 (75.2)
Lifetime having been drunk: n (%)	31 (40.3)	22 (32.4)	53 (36.6)
Maximum amount of alcohol consumed/occasion: mean (SD)	27.1 (28.0)	25.3 (34.3)	26.2 (31.0)

1 “enriched” family adversity index as proposed by Rutter and Quinton measuring the presence of 11 adverse family factors covering characteristics of the parents, the partnership, and the family environment during a period of one year prior to birth

2 obstetric adversity score counting the presence of 9 adverse conditions during pregnancy, delivery, and postnatal period such as preterm labor, asphyxia or seizures

### Drinking assessment

At age 15, adolescents were assessed for their alcohol consumption, using the Substance Use Questionnaire [21] and the Lifetime Drinking History Scale [22]). On average, these adolescents drank their first full glass of alcohol at 13.23±1.05 years. Two drinking variables were derived indicating the maximum amount of alcohol consumed per occasion, and lifetime prevalence of drunkenness.

### Mutation screening and promoter analysis

Search for single nucleotide polymorphisms in the *PIK3R1* gene was performed by sequencing 32 Caucasian DNA samples. 16 DNA pools consisting of an equimolar mixture of two DNA samples were prepared and used as PCR templates. For each gene, primers were chosen in order to amplify the regulatory domains, the exon-containing DNA fragments, including exon-intron boundaries. The PCRs were performed in a 15ul reaction mixture containing 25ng DNA. The list of the primers for each gene is available on the web site of the Centre National de Genotypage (CNG) (www.cng.fr). Sequencing reactions were performed using an ABI PRISM 3700 DNA Analyser (Applied Biosystems, Foster City, CA). Alignment of experimental results and identification of SNPs were done using the Genalys software developed by the CNG. Haplotype tagging SNPs (htSNPs) were selected to segregate haplotypes with a frequency >4.5%.

### SNP genotyping

High molecular weight genomic DNA was extracted from whole blood sample with standard salting out methods. Single nucleotide polymorphisms were genotyped using the TaqMan MGB biallelic discrimination system. Probes and primers were ordered from and automatically designed by Applied Biosystems using the Assay-by-Design product. PCR reactions were performed in Biometra T1 thermocyclers and fluorescence measured with an ABI Prism 7900HT sequence-detector end-point read. Genotyping errors and consistency across plates was checked using four common control samples per plate. Process and genotyping data were exported into an internal LIM System.

### Statistical analysis

The program package unphased [23] was used to test association between SNPs and alcohol drinking patterns. Tdtphase v.2.404, was used in family data analysis. This programme detects errors in Mendelian transmission patterns and if encountered a warning is printed and the pedigree is discarded. The number of transmissions of a particular allele (or haplotype) from a heterozygous parent to an affected child in a family is counted as well as the number of times, when this allele (haplotype) is not transmitted. These numbers are summed up over the whole sample of all nuclear families. Finally the equality of these two numbers is tested conducting a homogeneity (likelihood ratio) chi square test. Analyses were performed for the entire sample and separately for each gender. Estimation maximisation was enabled to also include uncertain haplotypes to increase the number of alleles included in the analysis. This is reflected by the uneven numbers in the transmitted versus untransmitted column. The cut off point for haplotype frequency was 1%. Haplotype association analyses were performed with COCAPHASE 2.3.5, using the expectation maximisation (EM) algorithm [23]. For differences between actual and expected frequencies of the htSNPs we employed Hardy–Weinberg equilibrium (HWE) equation as implemented in the DeFinetti Program (Strom Wienker. DeFinetti program online at http://ihg.gsf.de/cgi-bin/hw/hwa1.pl, 2005).

### In silico analyses

Phylogenetic analysis was performed by MULTIZ alignments using the PhastCons program [24] in the UCSC Genome Browser (http://genome.ucsc.edu). We assessed hypothetical function for SNPs using *in silico* analysis of transcription factor binding sites: both possible alleles of each SNP were tested for their binding capability to human transcription factors. Effect of SNP on transcription factor binding sites was analysed using Consite [25]. Putative alternative promoters were identified using the First-Exon and Promoter Prediction software (UCSC Genome Bioinformatics) [25].

## Results

### 1) Identification of SNPs and selection of htSNPs

**Figure 1:**
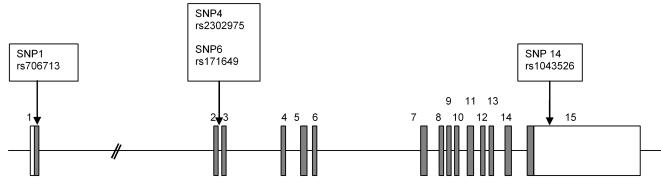
Map of the *PIK3R1* gene showing exons (boxes), introns (solid lines) and position of the 4 htSNPs that discriminate all haplotypes with a frequency of ≥4.5%. The graph is not to scale. Open boxes indicate untranslated regions, whereas shaded boxes display the coding regions.

To identify potentially functional genetic variations in *PIK3R1*, we sequenced the 5′ and 3′ regulatory domains, the exons and exon-intron boundaries of the gene. This led to identification of 22 SNPs. Only 14, with minor allele frequency above 6%, were included in subsequent analyses (Table 2). Analysis of haplotype distribution revealed that 4 htSNPs were sufficient to discriminate all haplotypes with a frequency of ≥4.5% (Table 3 and Figure 1). The genotypic frequencies of these SNPs were in Hardy-Weinberg equilibrium in these samples.

**Table 2:** PIk3R1 SNPs: position and minor allele frequencies

SNP #	SNP ID	Position on Chr 5	Localisation	Nucleotide variation	Amino acid change	Minor allele frequency
1	rs706713	67558478	Exon1	C to T	synonymous	0.217
2	rs706714	67558607	Intron1	A to C	-	0.182
3	rs2302974	67605147	Intron2	A to C	-	0.238
4	rs2302975	67605235	Intron2	A to G	-	0.440
5	rs2302976	67605358	Intron2	A to G	-	0.237
6	rs171649	67605502	Intron2	G to A	-	0.378
7	rs3730082	67605894	Intron3	G to A	-	0.313
8	rs16897620	67611100	Intron3	A to G	-	0.250
9	rs3815701	67611398	Intron4	A to G	-	0.250
10	novel	67611552	Intron4	A to C	-	0.150
11	rs3730089	67623904	Exon7	G to A	Met to Ile	0.219
12	rs895304	67628037	Intron14	G to A	-	0.211
13	rs3730073	67628049	Intron14	A to C	-	0.063
14	rs1043526	67630080	3′UTR	A to G	-	0.094

**Table 3:** Combination of tag SNP alleles defining each haplotype and frequencies

	Haplotypes					
	1	2	3	4	5	6
SNP #						
**1 (rs706713)**	**0**	**0**	**1**	**0**	**0**	**0**
2 (rs706714)	0	0	1	0	0	0
3 (rs2302974)	0	0	1	0	1	1
**4 (rs2302975)**	**0**	**1**	**1**	**0**	**1**	**1**
5 (rs2302976)	0	0	1	0	1	1
**6 (rs171649)**	**1**	**0**	**0**	**0**	**0**	**0**
7 (rs3730082)	0	1	0	0	0	0
8 (rs16897620)	0	0	1	0	1	1
9 (rs3815701)	0	0	1	0	1	1
10 (novel)	0	0	1	0	0	0
11 (rs3730089)	0	0	0	0	0	0
12 (rs895304)	0	0	0	0	0	0
13 (rs3730073)	0	0	0	0	0	0
**14 (rs1043526)**	**0**	**0**	**0**	**0**	**1**	**0**
Frequency	0.2726	0.1682	0.0625	0.0585	0.0469	0.0463

Haplotype tagging SNPs are indicated in bold. (0) indicates the allele with major frequency, (1) the allele with minor frequency.

### 2) Association of htSNPs with phenotypes of alcohol misuse in adolescents

#### -Individual *PIK3R1* SNPs

When we assessed the tagging SNPs for an association with specific alcohol drinking patterns in adolescents, we observed significant gender-specific overtransmission of the G-allele of SNP4 (rs2302975) and the G-allele of SNP14 (rs1043526) in adolescents displaying signs of alcohol misuse. In male adolescents, both htSNPs were associated with lifetime prevalence of drunkenness and with elevated maximum amounts of alcohol intake per occasion (≥16 g of alcohol per occasion), as measure for binge drinking pattern. These SNPs also associated with higher average levels of alcohol drinking per occasion in males. However, as this later phenotypic variable was highly correlated with the maximal amount of alcohol intake, we restricted our analysis on maximum levels of alcohol intake per occasion. SNP11 (rs3730089), that alters amino acid sequence of the encoded protein, did not show evidence of association. No association was observed between the htSNPs and alcohol drinking behaviour in females (Table 4).

**Table 4:** Association of *PIK3R1* SNPs with alcohol consumption patterns in adolescents (TDT)

		Males (n = 77)			Females (n = 68)			Total (n = 145)		
SNP	Alleles	T∶U	OR (95% CI)	P-value	T∶U	OR (95% CI)	P-value	T∶U	OR (95% CI)	P-value
*Lifetime prevalence drunkenness* a										
1 (rs706713)	T	12∶11	1.1152 (0.4464-2.7858)	0.8154	9∶10	0.8743 (0.3163-2.4167)	0.7955	21∶21	1 (0.50681.9732)	1.0000
	C	45∶46			35∶34			80∶80		
**4 (rs2302975)**	G	31∶15	3.3893 (1.5350-7.4836)	**0.0019**	20∶19	1.0965 (0.4727-2.5435)	0.8301	51∶34	2.02 (1.1426-3.5727)	**0.0148**
	A	25∶41			24∶25			49∶66		
6 (rs171649)	A	21∶30	0.5200 (0.2447-1.1052)	0.0870	17∶16	1.1050 (0.4601-2.6536)	0.8232	38∶46	0.7159 (0.4057-1.2633)	0.2479
	G	35∶26			25∶26			60∶52		
11 (rs3730089)	G	49∶51	0.6863 (0.2041-2.3078)	0.5403	35∶39	0.4986 (0.1525-1.6301)	0.2409	84∶90	0.5833 (0.2508-1.3569)	0.2053
	A	7∶5			9∶5			16∶10		
**14 (rs1043526)**	G	13∶5	3.0426 (1.0103-9.163)	**0.0379**	7∶10	0.6432 (0.2201-1.8794)	0.4169	20∶15	1.4127 (0.6788-2.9399)	0.3534
	A	47∶55			37∶34			84∶89		
*Maximum amount of drinking/occasion* b										
1 (rs706713)	T	12∶15	0.7538 (0.3211-1.77)	0.5154	9∶11	0.7801 (0.2930-2.0773)	0.6185	21∶26	0.7652 (0.4022-1.4559)	0.4137
	C	52∶49			43∶41			95∶90		
**4 (rs2302975)**	G	34∶17	3.1333 (1.4938-6.5724)	**0.0020**	19∶24	0.6717 (0.3065-1.4723)	0.319	53∶41	1.5389 (0.9080-2.6083)	0.1082
	A	30∶47			33∶28			63∶75		
6 (rs171649)	A	25∶32	0.641 (0.3178-1.2930)	0.2127	25∶19	1.6316 (0.7361-3.6163)	0.2262	50∶51	0.9651 (0.5722-1.6276)	0.8939
	G	39∶32			25∶31			64∶63		
11 (rs3730089)	G	56∶56	1 (0.3292-3.0380)	1	44∶47	0.5851 (0.1779-1.9245)	0.3718	100∶103	0.7767 (0.3464-1.7415)	0.5385
	A	7∶7			8∶5			15∶12		
**14 (rs1043526)**	G	14∶6	2.6923 (0.9651-7.5108)	**0.0494**	8∶15	0.4485 (0.1712-1.1749)	0.0960	22∶21	1.0585 (0.5464-2.0504)	0.8661
	A	52∶60			44∶37			96∶97		

T = transmitted, U untransmitted

a Subject has never been drunk vs. has been drunk

b Median split at 16 g alcohol

To detect a possible interaction between htSNP4 and htSNP14, we performed conditional TdT tests. Although this analysis confirmed the associations detected in the univariate analyses, no evidence for an interaction of the two genotypes could be observed. When htSNP4 conditioned on htSNP14, significant results were observed in males with regard to lifetime prevalence of drunkenness (P = 0.0136). However, when htSNP14 conditioned on htSNP4, no significant results were observed (P = 0.4324). This indicates that only htSNP4, but not htSNP14 contributes to the phenotypes observed.

#### -Haplotype analysis

We performed slide-window haplotype analysis for the analysed SNPs. Two-marker haplotype analysis showed that haplotypes SNP1C–SNP4G and SNP4G–SNP6G were significantly overtransmitted (P = 0.0243 and P = 0.0017, respectively) in adolescents that displayed life time prevalence of drunkenness and high amount of alcohol drinking (Table 5).

**Table 5:** Haplotypic association between *PIK3R1* and alcohol drinking patterns in male adolescents (n = 77)

Haplotypes	Global test	Individual haplotype test			
	P	Alleles (T∶U)	Chi^2^	Odds ratio	P
*Lifetime prevalence of drunkenness*					
1-4	**0.0088**	T-G (8.5∶2)	3.8280	1	0.0504
		T-A (2.5∶9)	3.5260	0.06615	0.0604
		C-G (22.5∶13)	5.0730	0.414	**0.0243**
		C-A (20.5∶30)	4.3950	0.1631	**0.0360**
4-6	**0.0044**	G-G (31∶15)	9.8590	1	**0.0017**
		A-A (20∶30)	3.7460	0.3326	0.0529
		A-G (3∶9)	3.5150	0.1613	0.0608
1-4-6	**0.0365**	T-G-G (8.4∶1.6)	4.8190	1	**0.0282**
		T-A-A (2.6∶6.4)	1.3250	0.07711	0.2496
		C-G-G (22.6∶13.4)	4.4120	0.3235	**0.0357**
		C-A-A (17.4∶23.6)	2.0050	0.1416	0.1567
		C-A-G (3∶6)	1.1100	0.09593	0.2920
*Maximum amount of drinking/occasion*					
1-4	**0.0019**	T-G (9.7∶3.2)	2.6840	1	0.1014
		T-A (1.3∶11.8)	8.8600	0.03714	**0.0029**
		C-G (24.3∶13.8)	5.8260	0.5785	**0.0158**
		C-A (26.7∶33.2)	2.1760	0.2641	0.1402
4-6	**0.0026**	G-G (34∶17)	9.7720	1	**0.0018**
		A-A (24∶32)	5.9697	0.375	**0.0145**
		A-G (04∶13)	5.775	0.1538	**0.0163**
1-4-6	**0.0029**	T-G-G (9.6∶2.8)	3.4000	1	0.0652
		T-A-A (1.4∶7.2)	3.4010	0.05763	0.0652
		T-A-G (00∶05)	7.1420	0	**0.0075**
		C-G-G (24.4∶14.2)	5.1310	0.5048	**0.0235**
		C-A-A (22.6∶24.8)	0.4075	0.267	0.5232
		C-A-G (04∶08)	1.5020	0.1467	0.2204
4-6-11	**0.0086**	G-G-G (04∶17)	4.6830	1	**0.0305**
		G-G-A (05∶00)	6.7420	−0.6071	**0.0094**
		A-A-G(24∶31)	3.3830	0.47	0.0659
		A-G-G (02∶07)	3.1630	0.1735	0.0753
		A-G-A (02∶05)	1.4070	0.2429	0.2356

While haplotype SNP1C–SNP4A (P = 0.036) was undertransmitted in children with lifetime prevalence of drunkenness, haplotypes SNP1T–SNP4A (P = 0.0029), SNP4A–SNP6A (P = 0.0145) and SNP4A–SNP6G (P = 0.0163) were undertransmitted in those drinking elevated quantities of alcohol. Three-marker haplotype analysis showed significant transmission distortion for the haplotype SNP1C-SNP4G-SNP6G (P = 0.0357). These results confirm our individual SNPs analysis and indicate that the region between SNP1 and SNP6 may be involved in conferring risk of alcohol misuse.

### 3) In silico analysis

**Figure 2:**
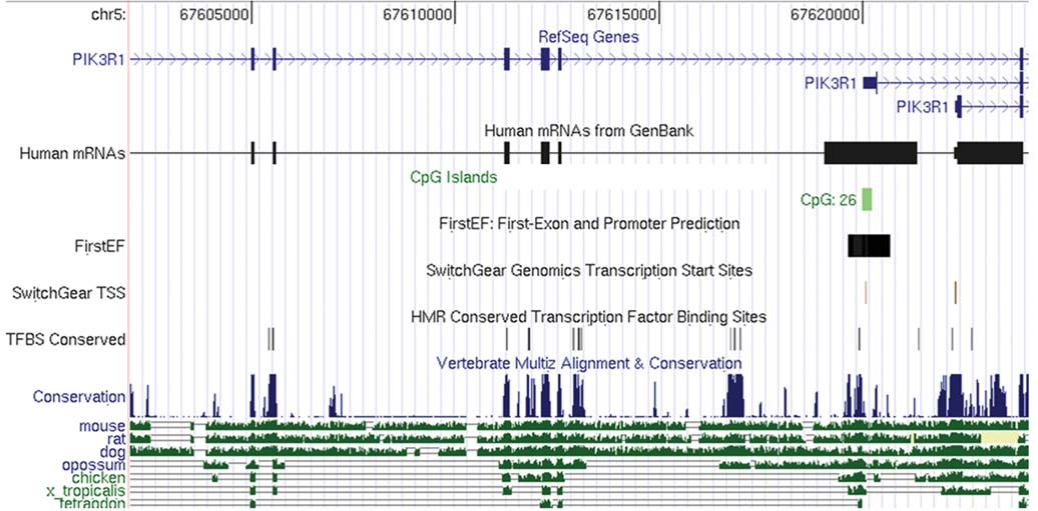
UCSC genome browser generated view of genomic region of *PIK3R1* including exons 2 to 7. Coding exons are represented by blocks connected by horizontal lines representing introns. The 5′ untranslated regions (UTRs) are displayed as thinner blocks on the leading ends of the aligning regions. Arrowheads on the connecting intron lines indicate the direction of transcription. Position of CpG islands, putative promoter and transcription start sites are indicated. Location of transcription factor binding sites conserved in the human/mouse/rat alignment is indicated. Conservation of binding sites in all 3 species is computed with the Transfac Matrix Database (v7.0). Evolutionary conservation in 17 vertebrates, including mammalian, amphibian, bird, and fish species was scored by the phastCons program following MULTIZ alignments. Conservation scores are displayed as wiggle, where the height reflects the size of the score. Pairwise alignments of each species to the human genome are displayed below as a “wiggle” that indicates alignment quality.

To investigate possible functional significance of the association identified, we performed in silico analysis of the genomic region surrounding SNP4, using the Human May 2004 Assembly of the UCSC Genome Browser (http://genome.ucsc.edu). The *PIK3R1* gene spans 86 Kb and encodes for three transcript variants, the shorter variants originating from the use of alternate first exons (Figure 2). The First-Exon and Promoter Prediction software [25] predicts two promoters for this gene. One promoter drives expression of the long isoform of PI3K while an alternative promoter located within intron 6, would be responsible for expression of the shorter isoforms. Presence of this alternative promoter suggests that SNP4 and other genetic variations located in its vicinity might influence its activity, thereby affecting the relative expression of specific PI3K isoforms. In higher organisms, transcription factor binding sites could appear in intronic or intergenic regions away from the transcription start site [26]. Figure 2 shows high phylogenic conservation of DNA regions within introns 3, 4 and 6, suggesting a conserved regulatory function. This is further supported by the overrepresentation of putative transcription factor binding sites, conserved in human, mice and rats, upstream of putative alternative transcription start sites. SNPs 3, 4, 5, 8 and 9 are in a genomic region displaying high linkage disequilibrium, as individuals carrying the G-allele of SNP4 often carry minor alleles at SNPs 3 5, 8 and 9, located in introns 2–4 (Table 3). A search for transcription factor binding sites using Consite [27] indicated that most of these SNPs alter putative transcription factor binding sites. Especially, the presence of an A or G allele at SNP8 determines the presence of a canonical E-box, binding site for basic Helix-Loop-Helix transcriptional regulators [28]. These results suggest that these SNPs are potentially functional.

## Discussion

In this study, we report evidence for association between the *PIK3R1* gene and signs of alcohol risk drinking behaviour in an adolescent sample. Evidence for association was obtained using single locus and haplotype-based analyses of 4 htSNPs that discriminated estimated haplotypes within *PIK3R1* with a frequency ≥4.5%. We demonstrated an association, mainly driven by htSNP4, with several signs of alcohol risk drinking behaviour in male adolescents, including lifetime prevalence of drunkenness and higher amount of drinking per occasion.

A crucial, clinically relevant aspect of our study is that lifetime prevalence of drunkenness and high amounts of alcohol consumed per occasion in the adolescent population that we analysed might reflect binge drinking patterns. Binge drinking is increasingly popular, especially among young people [29]. This form of drinking during adolescence is alarming not only because of the immediate consequences of acute alcohol intoxication, but also because early drinking is a strong predictor of later alcohol abuse and dependence and is associated with higher risk of alcohol-related injuries [1], [30]-[33]. Report from a large-scale study [31] indicates that the rates of lifetime alcohol dependence dropped from more than 40% among adolescents who started drinking at ages 14 or younger to approximately 10% among individuals who started drinking after age 20; the odds of dependence decreasing by 14% with each increasing year of age at onset of use. This was further refined by DeWit *et al.* [32] who observed a delay in progression to harm for the youngest drinkers (ages 10 and under) and identified the age range 11–14 as critical for mediating elevated risk of developing alcohol-related disorders. This observation was later confirmed and extended by McCarty *et al*. [1] who demonstrated that harmful drinking during adolescence was significantly associated with harmful drinking at ages 30 to 31 for men only, while binge drinking during adolescence was associated with binge drinking at ages 30 to 31 for both men and women.

Of particular interest was the gender-specific association, which was observed in males but not females. Gender differences in alcohol consumption have long been recognized, and attempts to explain the much higher rate of alcohol use disorders among men compared with women have been published [for a recent review, see 34]. This led to the proposal that besides genetic risk-factors, negative physiological effects of alcohol and psychosocial factors influence alcohol drinking behaviour in women [34]. Yet, a link between PI3K and sex steroids has been found, as nongenomic functions of androgens [35] and estrogens [36] are mediated by ligand-induced complex formation between their respective receptor and PI3K. However, ability of estrogens to activate PI3K seems to be considerably higher than that of androgens [36]. This difference may account for the gender-specific association found in our study.

Is there a role for PIK in addiction? Drugs of abuse, including alcohol, are thought to affect molecular and cellular mechanisms that underlie long-term associative memories, like activity-induced long-term potentiation (LTP) and long-term depression (LTD) of synaptic transmission [37]. PI3K activity is necessary for synaptic transmission and regulates both LTP [38], [39] and LTD [13], [16], [40]. Animal experiments demonstrated that PI3K is required for the expression but not for the induction or the maintenance of LTP in the hippocampus [41] and for the expression of behavioral sensitization of rats to cocaine [18], establishing a similarity between the role of PI3K in LTP and in cocaine-induced behavioural sensitisation. PIK has been shown to regulate synaptic plasticity by controlling exocytosis of AMPA-type glutamate receptors [15] and by coupling group I mGluRs to translation initiation [16]. Thus, a role for PI3K in alcoholism might be inferred from its involvement in the glutamatergic transmission. Besides, a role for PI3K in addiction is further suggested by its role in neurotrophin-mediated potentiation and transmitter release [38]. This hypothesis is supported by a recent study showing association of antisocial alcohol dependence with SNPs within the human neurotrophin receptor TrkB gene [42].

Our SNP analysis was limited to the regulatory regions of the gene, including exon-intron boundaries and led to the identification of a previously unknown SNP. The functional impact of these SNPs on *PIK3R1* regulation and activity is not known, however hypothesis as to the molecular mechanisms that could mediate their effect can be generated. For example, htSNP4 and most of the SNPs in its vicinity alter putative binding sites of transcription factors that might influence transcriptional activity of this gene. Specifically, SNP8 which is in strong linkage disequilibrium with SNP4 creates a canonical E-box, binding site for basic helix-loop-helix transcriptional regulators. Members of this family of transcription factors play key roles in the development of organisms ranging from yeast to humans. Several are widely expressed, while others including the neurogenin subfamily that play an important role in neurogenesis show a tissue-restricted pattern [43]–[45]. Regulatory elements that significantly contribute to gene expression are present in intronic regions of several genes [26], including the human serotonin transporter gene [46], [47]. Thus, it is not unreasonable to propose that the association reported in this study reflect genotype-specific transcriptional activity of the *PIK3R1* gene. Alternatively, the genetic variation mediating the association might affect splicing of the *PI3KR1* transcript [48], or expression of associated regulatory non-coding RNAs [49].

Taken together, our results indicate that variations in the *PIK3R1* gene are associated with multiple aspects of alcohol risk drinking behaviour in male adolescents, suggesting a relevance of *PIK3R1* genotypes for early onset of alcoholism in humans. It is noteworthy that the adolescents in our study had their first drink at age 13.23 +/− 1.05 years on average when assessed for alcohol consumption at age 15, an age which might be too early for complete development of risky alcohol drinking behaviour. Follow up studies including drinking behaviour of these individuals later in adolescence and early adulthood will be of great interest to fully assess drinking behaviour. As environmental components like parental substance use, parenting practice and peer influence might play a significant role in the development of alcohol use disorders [50], inclusion of these informations as covariables in those studies will help monitor possible gene×environment interactions. In addition, a limitation of our work is its relatively small sample size, which prevents powerful statistical analysis. Experiments involving the replication of our findings in other populations, identification and characterisation of the functional variants conferring risk of alcohol misuse will have to be carried out to definitely gauge how alterations in *PI3KR1* impacts alcohol response-related behaviours.

## References

[1] McCarty CA, Ebel BE, Garrison MM, DiGiuseppe DL, Christakis DA (2004). Continuity of binge and harmful drinking from late adolescence to early adulthood.. Pediatrics.

[2] Tyndale RF (2003). Genetics of alcohol and tobacco use in humans.. Ann Med.

[3] Crabbe JC (2002). Genetic contributions to addiction.. Annu Rev Psychol.

[4] Higuchi S, Matsushita S, Kashima H (2006). New findings on the genetic influences on alcohol use and dependence.. Curr Opin Psychiatry.

[5] Uhl GR, Drgon T, Johnson C, Fatusin OO, Liu QR (2008). “Higher order” addiction molecular genetics: Convergent data from genome-wide association in humans and mice.. Biochem Pharmacol.

[6] Tsai G, Coyle JT (1998). The role of glutamatergic neurotransmission in the pathophysiology of alcoholism.. Annu Rev Med.

[7] Dodd PR, Beckmann AM, Davidson MS, Wilce PA (2000). Glutamate-mediated transmission, alcohol, and alcoholism.. Neurochem Int.

[8] Backstrom P, Bachteler D, Koch S, Hyytia P, Spanagel R (2004). mGluR5 antagonist MPEP reduces ethanol-seeking and relapse behavior.. Neuropsychopharmacology.

[9] Schroeder JP, Overstreet DH, Hodge CW (2005). The mGluR5 antagonist MPEP decreases operant ethanol self-administration during maintenance and after repeated alcohol deprivations in alcohol-preferring (P) rats.. Psychopharmacology (Berl).

[10] Olive MF, McGeehan AJ, Kinder JR, McMahon T, Hodge CW (2005). The mGluR5 antagonist 6-methyl-2-(phenylethynyl)pyridine decreases ethanol consumption via a protein kinase C epsilon-dependent mechanism.. Mol Pharmacol.

[11] Cowen MS, Djouma E, Lawrence AJ (2005). The metabotropic glutamate 5 receptor antagonist 3-[(2-methyl-1,3-thiazol-4-yl)ethynyl]-pyridine reduces ethanol self-administration in multiple strains of alcohol-preferring rats and regulates olfactory glutamatergic systems.. J Pharmacol Exp Ther.

[12] Hodge CW, Miles MF, Sharko AC, Stevenson RA, Hillmann JR (2006). The mGluR5 antagonist MPEP selectively inhibits the onset and maintenance of ethanol self-administration in C57BL/6J mice.. Psychopharmacology (Berl).

[13] Daw MI, Bortolotto ZA, Saulle E, Zaman S, Collingridge GL (2002). Phosphatidylinositol 3 kinase regulates synapse specificity of hippocampal long-term depression.. Nat Neurosci.

[14] Perkinton MS, Ip JK, Wood GL, Crossthwaite AJ, Williams RJ (2002). Phosphatidylinositol 3-kinase is a central mediator of NMDA receptor signalling to MAP kinase (Erk1/2), Akt/PKB and CREB in striatal neurones.. J Neurochem.

[15] Man HY, Wang Q, Lu WY, Ju W, Ahmadian G (2003). Activation of PI3-kinase is required for AMPA receptor insertion during LTP of mEPSCs in cultured hippocampal neurons.. Neuron.

[16] Hou L, Klann E (2004). Activation of the phosphoinositide 3-kinase-Akt-mammalian target of rapamycin signaling pathway is required for metabotropic glutamate receptor-dependent long-term depression.. J Neurosci.

[17] Gong R, Park CS, Abbassi NR, Tang SJ (2006). Roles of glutamate receptors and the mammalian target of rapamycin (mTOR) signaling pathway in activity-dependent dendritic protein synthesis in hippocampal neurons.. J Biol Chem.

[18] Izzo E, Martin-Fardon R, Koob GF, Weiss F, Sanna PP (2002). Neural plasticity and addiction: PI3-kinase and cocaine behavioral sensitization.. Nat Neurosci.

[19] Corl AB, Rodan AR, Heberlein U (2005). Insulin signaling in the nervous system regulates ethanol intoxication in Drosophila melanogaster.. Nat Neurosci.

[20] Laucht M, Esser G, Baving L, Gerhold M, Hoesch I (2000). Behavioral sequelae of perinatal insults and early family adversity at 8 years of age.. J Am Acad Child Adolesc Psychiatry.

[21] Müller R, Abbet JP (1991). [Changing trends in the consumption of legal and illegal drugs by 11-16-year-old adolescent pupils. Findings from a study conducted under the auspices of the WHO Europe]..

[22] Skinner HA, Sheu WJ (1982). Reliability of alcohol use indices. The Lifetime Drinking History and the MAST.. J Stud Alcohol.

[23] Dudbridge F (2003). Pedigree disequilibrium tests for multilocus haplotypes.. Genet Epidemiol.

[24] Siepel A, Bejerano G, Pedersen JS, Hinrichs AS, Hou M (2005). Evolutionarily conserved elements in vertebrate, insect, worm, and yeast genomes.. Genome Res.

[25] Davuluri RV, Grosse I, Zhang MQ (2001). Computational identification of promoters and first exons in the human genome.. Nat Genet.

[26] Long F, Liu H, Hahn C, Sumazin P, Zhang MQ (2004). Genome-wide prediction and analysis of function-specific transcription factor binding sites.. In Silico Biol.

[27] Sandelin A, Wasserman WW, Lenhard B (2004). ConSite: web-based prediction of regulatory elements using cross-species comparison.. Nucleic Acids Res.

[28] Massari ME, Murre C (2000). Helix-loop-helix proteins: regulators of transcription in eucaryotic organisms.. Mol Cell Biol.

[29] Pincock S (2003). Binge drinking on rise in UK and elsewhere. Government report shows increases in alcohol consumption, cirrhosis, and premature deaths.. Lancet.

[30] Bonomo YA, Bowes G, Coffey C, Carlin JB, Patton GC (2004). Teenage drinking and the onset of alcohol dependence: a cohort study over seven years.. Addiction.

[31] Grant BF, Dawson DA (1997). Age at onset of alcohol use and its association with DSM-IV alcohol abuse and dependence: results from the National Longitudinal Alcohol Epidemiologic Survey.. J Subst Abuse.

[32] DeWit DJ, Adlaf EM, Offord DR, Ogborne AC (2000). Age at first alcohol use: a risk factor for the development of alcohol disorders.. Am J Psychiatry.

[33] Hingson R, Heeren T, Zakocs R, Winter M, Wechsler H (2003). Age of first intoxication, heavy drinking, driving after drinking and risk of unintentional injury among U.S. college students.. J Stud Alcohol.

[34] Nolen-Hoeksema S, Hilt L (2006). Possible contributors to the gender differences in alcohol use and problems.. J Gen Psychol.

[35] Sun M, Yang L, Feldman RI, Sun XM, Bhalla KN (2003). Activation of phosphatidylinositol 3-kinase/Akt pathway by androgen through interaction of p85alpha, androgen receptor, and Src.. J Biol Chem.

[36] Simoncini T, Hafezi-Moghadam A, Brazil DP, Ley K, Chin WW (2000). Interaction of oestrogen receptor with the regulatory subunit of phosphatidylinositol-3-OH kinase.. Nature.

[37] Hyman SE, Malenka RC, Nestler EJ (2006). Neural mechanisms of addiction: the role of reward-related learning and memory.. Annu Rev Neurosci.

[38] Yang F, He X, Feng L, Mizuno K, Liu XW (2001). PI-3 kinase and IP3 are both necessary and sufficient to mediate NT3-induced synaptic potentiation.. Nat Neurosci.

[39] Kelly A, Lynch MA (2000). Long-term potentiation in dentate gyrus of the rat is inhibited by the phosphoinositide 3-kinase inhibitor, wortmannin.. Neuropharmacology.

[40] Huang CC, Lee CC, Hsu KS (2004). An investigation into signal transduction mechanisms involved in insulin-induced long-term depression in the CA1 region of the hippocampus.. J Neurochem.

[41] Sanna PP, Cammalleri M, Berton F, Simpson C, Lutjens R (2002). Phosphatidylinositol 3-kinase is required for the expression but not for the induction or the maintenance of long-term potentiation in the hippocampal CA1 region.. J Neurosci.

[42] Xu K, Anderson TR, Neyer KM, Lamparella N, Jenkins G (2007). Nucleotide sequence variation within the human tyrosine kinase B neurotrophin receptor gene: association with antisocial alcohol dependence.. Pharmacogenomics J.

[43] Urbanek P, Wang ZQ, Fetka I, Wagner EF, Busslinger M (1994). Complete block of early B cell differentiation and altered patterning of the posterior midbrain in mice lacking Pax5/BSAP.. Cell.

[44] Sheng HZ, Zhadanov AB, Mosinger B, Fujii T, Bertuzzi S (1996). Specification of pituitary cell lineages by the LIM homeobox gene Lhx3.. Science.

[45] Sharma K, Sheng HZ, Lettieri K, Li H, Karavanov A (1998). LIM homeodomain factors Lhx3 and Lhx4 assign subtype identities for motor neurons.. Cell.

[46] MacKenzie A, Quinn J (1999). A serotonin transporter gene intron 2 polymorphic region, correlated with affective disorders, has allele-dependent differential enhancer-like properties in the mouse embryo.. Proc Natl Acad Sci U S A.

[47] Fiskerstrand CE, Lovejoy EA, Quinn JP (1999). An intronic polymorphic domain often associated with susceptibility to affective disorders has allele dependent differential enhancer activity in embryonic stem cells.. FEBS Lett.

[48] Pagani F, Baralle FE (2004). Genomic variants in exons and introns: identifying the splicing spoilers.. Nat Rev Genet.

[49] Mattick JS, Makunin IV (2006). Non-coding RNA.. Hum Mol Genet.

[50] Sher KJ, Grekin ER, Williams NA (2005). The development of alcohol use disorders.. Annu Rev Clin Psychol.

